# Thymic Atrophy and Immune Dysregulation in Infants with Complex Congenital Heart Disease

**DOI:** 10.1007/s10875-024-01662-4

**Published:** 2024-02-23

**Authors:** Sarah-Jolan Bremer, Annika Boxnick, Laura Glau, Daniel Biermann, Simon A. Joosse, Friederike Thiele, Elena Billeb, Jonathan May, Manuela Kolster, Romy Hackbusch, Mats Ingmar Fortmann, Rainer Kozlik-Feldmann, Michael Hübler, Eva Tolosa, Jörg Siegmar Sachweh, Anna Gieras

**Affiliations:** 1 Department of Immunology, University Medical Center Hamburg-Eppendorf, N27, Martinistraße 52, 20246 Hamburg, Germany; 2 University Children’s Research, UCR@Kinder-UKE, University Medical Center Hamburg-Eppendorf, Hamburg, Germany; 3 Congenital and Pediatric Heart Surgery, Children’s Heart Clinic, University Heart & Vascular Center Hamburg, University Medical Center Hamburg-Eppendorf, Hamburg, Germany; 4 German Centre for Cardiovascular Research (DZHK), Partner Site Hamburg/Kiel/Lübeck, Hamburg, Germany; 5 Department of Tumor Biology, University Medical Center Hamburg-Eppendorf, Hamburg, Germany; 6 Mildred Scheel Cancer Career Center HaTriCS4, University, Medical Center Hamburg-Eppendorf, Hamburg, Germany; 7 Department of Pediatrics, University of Lübeck, Lübeck, Germany; 8 Department of Pediatric Cardiology, University Heart & Vascular Center Hamburg, University Medical Center Hamburg-Eppendorf, Hamburg, Germany

**Keywords:** Congenital heart disease (CHD), Thymus, T-cell development, Thymic atrophy, Recent thymic emigrants (RTE)

## Abstract

Congenital heart disease (CHD) is the most common birth defect, and up to 50% of infants with CHD require cardiovascular surgery early in life. Current clinical practice often involves thymus resection during cardiac surgery, detrimentally affecting T-cell immunity. However, epidemiological data indicate that CHD patients face an elevated risk for infections and immune-mediated diseases, independent of thymectomy. Hence, we examined whether the cardiac defect impacts thymus function in individuals with CHD. We investigated thymocyte development in 58 infants categorized by CHD complexity. To assess the relationship between CHD complexity and thymic function, we analyzed T-cell development, thymic output, and biomarkers linked to cardiac defects, stress, or inflammation. Patients with highly complex CHD exhibit thymic atrophy, resulting in low frequencies of recent thymic emigrants in peripheral blood, even prior to thymectomy. Elevated plasma cortisol levels were detected in all CHD patients, while high NT-proBNP and IL-6 levels were associated with thymic atrophy. Our findings reveal an association between complex CHD and thymic atrophy, resulting in reduced thymic output. Consequently, thymus preservation during cardiovascular surgery could significantly enhance immune function and the long-term health of CHD patients.

**Supplementary Information:**

The online version contains supplementary material available at 10.1007/s10875-024-01662-4.

## Introduction

The thymus plays a crucial role in establishing a functional adaptive immune response. The specialized thymic microenvironment supports the development of bone marrow–derived lymphoid progenitors into mature T cells with a broad but self-tolerant repertoire [1, 2]. The thymus reaches its maximum size at puberty (35–40 g), and thereafter it undergoes involution [1]. Age-related involution, also described as immunosenescence, is characterized by a decrease in thymus size and thymic output and comes along with reduced numbers of recent thymic emigrants (RTE) in the periphery [1, 3]. Besides age, a reduction of thymic output can be caused by various other factors including infections, malnutrition, certain diseases, or surgical interventions, such as partial or total thymectomy [3–8].

Congenital heart disease (CHD) is the most common birth defect worldwide and affects around 1% of newborns [9]. CHDs are defined as malformations of the heart and/or thoracic vessels and represent a highly heterogeneous group of heart anomalies that range from mild anatomical and pathophysiological abnormalities (e.g., atrial septal defect, ASD) to complex cardiovascular defects such as transposition of the great arteries (TGA) [10, 11]. Certain forms of CHD are classified as cyanotic and characterized by reduced systemic oxygen saturation due to the disruption of the normal blood flow [12]. Depending on the complexity of the heart defect, in approximately up to 50% of CHD patients cardiovascular surgery is needed within their first year of life [11]. Furthermore, CHD may be associated with genetic syndromes, including DiGeorge syndrome (DGS) or Down syndrome, which often come along with immune dysfunction and may further impact the health of individuals with CHD [13–15]. Despite advances in medical treatment, children with CHD suffer from frequent infections and face an elevated risk of morbidity and mortality during infancy [16, 17]. Previous studies have shown that reduced T-cell receptor excision circles (TREC), a marker for reduced thymic output, might be linked to non-immunologic conditions, among them cardiac defects [18–22].

In this study, we explored the impact of the heart defect on thymic function in pediatric CHD patients. We performed a comprehensive phenotypic analysis of thymocytes at different stages of T-cell development, measured RTE, and analyzed biomarkers that are associated with cardiac defects, stress, or inflammation in patients with different forms of CHD.

## Materials and Methods

### Patients

Thymic tissue and blood samples were obtained from 58 children undergoing corrective or palliative cardiac surgery in our institution between 2018 and 2022. A detailed description of patient characteristics is displayed in Table 1. Clinical variables including sex, age, CHD primary diagnosis, gestational age at birth, severe comorbidities, and NT-proBNP and Troponin T levels were assessed. The cardiac diagnoses were grouped into six CHD primary disease groups, namely, ASD, ventricular septal defect (VSD), complete atrioventricular septal defect (cAVSD), tetralogy of Fallot (ToF), TGA, and hypoplastic left heart syndrome (HLHS)/interrupted aortic arch (IAA) (or severe hypoplastic aortic arch). Ten patients presented with trisomy 21 and one patient with trisomy 18. When DiGeorge syndrome or DiGeorge-like syndrome was genetically confirmed at time of surgery, those children were excluded from the cohort. Genetic testing was performed in patients exhibiting a combination of typical clinical manifestations (e.g., CHD, hypocalcemia, hypothyroidism, thrombocytopenia, feeding difficulties, developmental delays, microcephaly, idiopathic seizures, hypotonia, unique facial characteristics, renal abnormalities, laryngo-tracheo-esophageal abnormalities, skeletal differences, chronic infection, or intrauterine growth retardation [14]). Thymic tissue of patients with trisomy 21 was analyzed, and the corresponding data were included in the supplementary materials. However, these data were excluded from CHD subgroup analysis to minimize potential bias. Two children with CHD that did not match the CHD primary disease groups mentioned in this paper were excluded.

**Table 1:** Patient characteristics

	*n*	Lymphocytes in peripheral blood [10^9^/l] (*n*=43)		NT-proBNP [ng/l] (*n*=46)		Troponin T [pg/ml] (*n*=21)	
		Count	(% of WBC)	Mean	(SD)	Mean	(SD)
All	46	3.26	(43.12)	14762	(16728)	49	(61)
Age							
< 30 days	23	2.70	(37.17)	27428	(15088)	90	(80)
30–180 days	8	3.20	(50.14)	4211	(4244)	43	(41)
> 180 days	15	4.06	(48.18)	969	(1298)	16	(8)
Sex							
Female	15	3.55	(43.49)	17731	(18112)	46	(51)
Male	31	3.10	(42.92)	13326	(16128)	50	(64)
CHD primary diagnosis							
ASD	4	4.53	(44.58)	1635	(2174)	10	n/a
VSD	9	3.65	(44.46)	1397	(2580)	30	(34)
cAVSD	5	3.24	(46.24)	4636	(4725)	17	(5)
ToF	6	3.73	(59.73)	1727	(14)	23	(1)
TGA	14	2.74	(42.08)	23095	(8846)	90	(78)
HLHS/IAA	8	2.66	(28.21)	37885	(18224)	90	(102)
Cyanosis							
No	18	3.74	(45.01)	2350	(3370)	24	(27)
Yes	28	2.95	(41.88)	22742	(17043)	77	(77)
Prematurity							
No	34	3.41	(42.14)	16817	(17751)	60	(70)
Yes	12	2.87	(45.66)	8940	(12222)	22	(8)

*CHD*, congenital heart disease; *ASD*, atrial septal defect; *VSD*, ventricular septal defect; *cAVSD*, complete atrioventricular septal defect; *ToF*, tetralogy of Fallot; *TGA*, transposition of the great arteries; *HLHS*, hypoplastic left heart syndrome; *IAA*, interrupted aortic arch; *NT-proBNP*, N-terminal pro-B-type natriuretic peptide; *SD*, standard deviation; *WBC*, white blood cells

### Sample Origin and Tissue Preparation

Thymic tissue was removed as part of the routine during heart surgery; no thymic tissue was removed for study reasons only. Thymic tissue was mechanically disrupted and subsequently filtered through a 70-μm nylon mesh to obtain a thymocyte suspension. Blood samples in EDTA tubes were taken during induction of anesthesia. A complete blood count test was performed using CELL-DYN Emerald Cell Counter (Abbott, Abbott Park (IL), USA). Subsequently, plasma was isolated. Plasma samples from children without CHD between 2 and 403 days of age (control group) were kindly provided by Dr. Mats Ingmar Fortmann from UKSH (Campus Lübeck).

### Immunophenotyping

Thymocytes were stained with following fluorochrome-conjugated antibodies: anti-CD1a FITC (clone: HI149), anti-CD3 BV650 (clone: OKT3), anti-CD4 PE-Dazzle594 (clone: RPA-T4), anti-CD7 AF700 (clone: M-T701), anti-CD8a BV785 (clone: RPA-T8), anti-CD25 BV421 (clone: BC96), anti-CD28 PE-Cy7 (clone: CD28.2), anti-CD34 PE (clone: 563), anti-CD45 BV510 (clone: HI30), anti-CD45RA BV711 (clone: HI100), anti-CD69 PerCP-Cy5.5 (clone: FN50), anti-Tγδ BV605 (clone: 11F2), Annexin V AF647, and for dead cell exclusion AF750 NHS Ester. For staining of whole blood to analyze RTE, the following antibodies were used: anti-CD3 BV510 (clone: OKT3), anti-CD4 AF700 (clone: OKT4), anti-CD8a BV605 (clone: RPA-T8), anti-CD25 BV421 (clone: BC96), anti-CD31 APC-Cy7 (clone: WM59), anti-CD45RA PE-Dazzle594 (clone: HI100), and anti-CD127 BV650 (clone: A019D5). Staining of thymocytes and whole blood was performed for 30 min at 4 °C and room temperature, respectively. For whole blood staining, red blood cells were lysed subsequently. The samples were washed in FACS buffer and resuspended in this same buffer prior to analysis. Acquisition of data was performed on an LSR Fortessa (BD Biosciences). Prior to analysis, a spillover spreading matrix was produced, antibodies were titrated, PMT voltages were optimized, and a compensation matrix was calculated. The FlowJo plugin FlowAI [23] was used to clean the flow cytometry files from artefactual events. The panel for the analysis of thymocytes is published as OMIP 073 [24].

### Cortisol ELISA

Morning plasma cortisol levels were measured by enzyme-linked immunosorbent assay (ELISA) (Cortisol Competitive ELISA Kit; Thermo Fisher Scientific) according to the manufacturer’s instructions (CHD *n* = 30, control group *n* = 21). Ninety-six-well plates coated with goat anti-mouse IgG were incubated with diluted plasma samples, assay buffer, cortisol conjugate, and cortisol antibody for 1 h at room temperature. After washing, the chromogen TMB substrate was incubated for further 30 min at room temperature. Absorbance was measured at 405 nm after stopping the colorimetric reaction in a multilabel plate reader (Victor3; PerkinElmer, MA, USA).

### Serum Proteomic Analysis

Inflammatory mediators were assessed in plasma samples (*n* = 40) using the Olink Target 96 Inflammation (v.3023) platform with proximity extension assay (PEA). Samples were prepared according to Olink’s instructions. In this assay, biomarker-specific antibodies tagged with oligonucleotides are detected by quantitative real-time polymerase chain reaction (qPCR). Cycle threshold values from qPCR are translated into a relative quantification unit and reported as normalized protein expression (NPX) values. Quality control and normalization were performed by Olink.

### Data Analysis

FlowJo Software versions 10.6.2 and 10.8.1 (FlowJo, LLC, Ashland, USA) were used for analysis of flow cytometry data. Data were exported as FCS files from FACSDiva version 9.0.1 for subsequent cleaning by FlowAI [23] and manual gating in FlowJo Software using the gating strategies published in OMIP 073 [24] for analysis of thymocytes. Dimensionality reduction of the cell population frequencies of all samples was performed in R (version 3.6.1) [25] using the R-package “umap” [26] with default parameters. The resulting uniform manifold approximation and projection (UMAP) representation was colored by CHD, age, sex, NT-proBNP, Troponin T, and IL-6. Cell population frequencies were also used as input for the hierarchical clustering function “hclust” from the R-package “stats,” used with agglomeration method “ward.D2” to find groups of samples in the data. Using the R-package “pheatmap” [27], a heatmap representation of the cell population frequencies was created, integrating the hierarchical clustering method “ward.D2” for samples and cell populations. In addition, dimensionality reduction of live, CD45^+^ cells was performed in R. One exemplary FCS file from each of the six primary disease groups (ASD, VSD, cAVSD, ToF, TGA, HLHS/IAA) was selected and randomly subsampled to 100,000 cells per file. To account for inter-sample acquisition variability, the algorithm “gaussNorm” from the R-package “flowStats” [28] was applied to the files. Subsampled and normalized files were concatenated and subjected to dimensionality reduction using the R-package “umap” with default parameters. The markers Annexin V (Alexa Fluor 647-A) and live/dead (Alexa Fluor 750-A) were excluded because only living cells were included in the analysis. After dimensionality reduction, events were segregated according to the different CHD groups and the resulting UMAP representations were colored by expression of surface markers included in the panel and by cell density. In addition, manually gated cell populations were overlaid onto the UMAP embedding to visualize their locations on the UMAP representation. For figure production, INKSCAPE (https://inkscape.org/de/) and BioRender (https://biorender.com/) were used.

### Statistics

GraphPad Prism versions 6.07 and 9.3.1 for Windows (GraphPad Software, San Diego, California USA, www.graphpad.com) were used for statistical analysis. First, data were analyzed for normal distribution. For linear regression analysis, data were not assumed to be sampled from Gaussian distribution and nonparametric Spearman correlation was computed. The 95% confidence interval (CI) of linear regression analysis is indicated in the plot by dashed lines. In these plots, *p* values (two-tailed) are indicated irrespectively of significance. For comparison between primary disease groups, statistical analysis was performed with one-way ANOVA followed by Bonferroni correction if data passed the normality test and with Kruskal–Wallis test and Dunn’s multiple comparisons test if data did not pass the normality test. Comparison between acyanotic and cyanotic CHD groups was performed using Student’s *t*-test if data passed normality test and Mann–Whitney test if data did not pass the normality test. Box plots show the median with standard deviation. *P* values are indicated in case of significance (*p < 0.05, **p < 0.01, ***p < 0.001, ****p < 0.0001).

## Results

### Patient Characteristics

Our cohort includes 46 children with CHD who underwent corrective or palliative cardiac surgery (for patient characteristics, see Table 1). Fifteen patients (33%) were female; 31 patients (67%) were male. The age of the patients at time of surgery ranged between 2 and 363 days with a mean age of 103 days. Patients were distributed to age groups as follows: 23 neonates below 30 days of age (50%), 8 infants from 30 to 180 days of age (17%), and 15 infants above 180 days of age (33%). Twelve patients (26%) were born preterm. In addition, we analyzed the thymic tissue, plasma, and peripheral blood mononuclear cells of 10 CHD patients with trisomy 21 and two CHD patients with severe postnatal infections.

Patients were categorized according to their primary CHD. ASD and VSD are common, less severe, and usually acyanotic malformations of the heart. Symptoms and management depend on size and structure of the defect [29, 30]. Infants that were included in this study underwent surgical repair to prevent potentially lethal complications like pulmonary hypertension. cAVSD is a heart defect with a common atrioventricular junction, and newborns may present with mild central cyanosis [31]. The most common form of cyanotic heart defect is ToF. After surgical repair, most patients have an essentially normal childhood [32]. TGA and HLHS are also classified as cyanotic heart defects and are seen as the most complex forms of CHD. Patients with complete TGA have a systemic and pulmonary circulation running in parallel and require surgical repair utilizing the arterial switch procedure [33]. Patients with HLHS have a functionally univentricular heart. Before conversion to the Fontan circulation was available, children with HLHS did not survive longer than a few weeks [34]. IAA is a severe malformation of the heart and leads to ischemia of the lower body as soon as ductal closure occurs within the first days of life. IAA was associated with high mortality before adequate treatment was available [35]. For our analysis, HLHS and IAA were grouped as they are a form of “left heart hypoplasia” and require early surgical palliation or repair, respectively, in the first month of life. CHD is often associated with chromosomal abnormalities such as aneuploidies, with trisomy 21 being the most common [36]. In our study population, 10 patients had trisomy 21 and were distributed as follows: five patients with cAVSD, two with VSD, one with ASD, one with ToF, and one with HLHS/IAA. One patient had trisomy 18 and presented with ToF. Two patients presented with sepsis and necrotizing enterocolitis (NEC), respectively. In the rest of the cohort, ASD was diagnosed in four patients, VSD in nine patients, cAVSD in five patients, ToF in six patients, TGA in 14 patients, and HLHS/IAA in eight patients, meaning that 18 patients (39%) presented with acyanotic CHD and 28 patients (61%) with cyanotic CHD.

### Complex CHD Is Associated with Thymic Atrophy

**Fig. 1:**
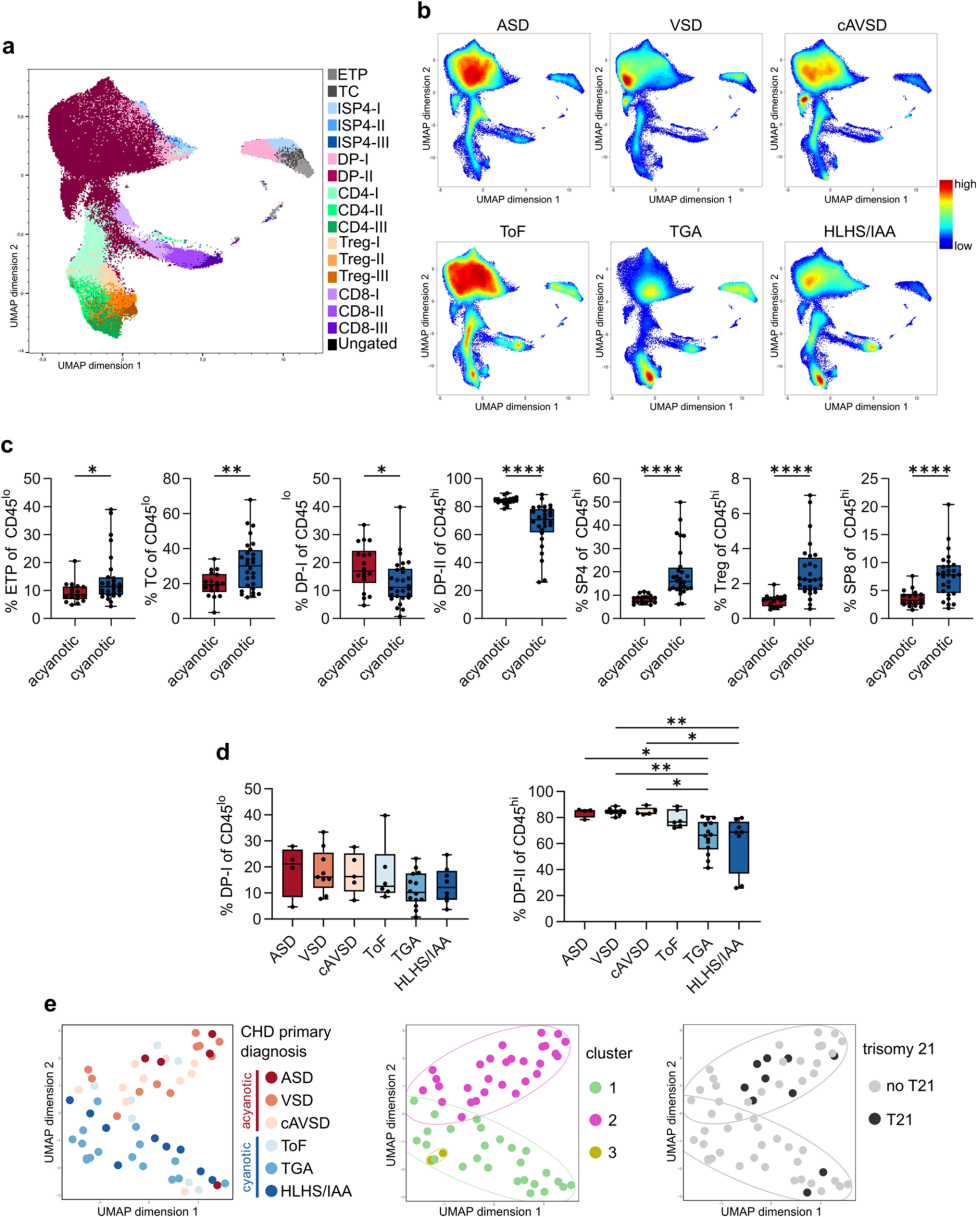
Complex CHD is associated with thymic atrophy. (**a**) UMAP plot illustrating the main thymocyte subpopulations identified by manual gating. (**b**) UMAP plots of exemplary thymi for the CHD primary disease groups. For the group HLHS/IAA, a sample from a patient with IAA is depicted. Cell density is depicted by color. (**c**) Comparison of thymocyte subpopulations in acyanotic and cyanotic CHD. Statistical analysis was performed with Mann–Whitney test. Data were obtained from the cohort presented in Tables 1 and 2 (*n* = 46). (**d**) Comparison of the thymocyte subsets DP-I and DP-II between the CHD groups. Statistical analysis was performed with one-way ANOVA and Bonferroni’s multiple comparisons test and Kruskal–Wallis test and Dunn’s multiple comparisons test, respectively, and is indicated in case of significance (*n* = 46). (**e**) UMAP plot showing thymocyte signatures in relation to CHD primary disease group and cyanosis as well as UMAP showing clusters obtained by hierarchical clustering and UMAP showing thymocyte signatures in relation to trisomy 21. UMAP was calculated on the data summarized in “Supporting data values” (*n* = 58 donors and 21 thymocyte subsets). Each dot represents one donor

To find out whether T-cell development occurs normally in the different types of CHD, we performed in-depth profiling of the thymocyte subpopulations in each of the 58 CHD patients. Using expert manual gating, we identified and analyzed the frequencies of 21 thymocyte subpopulations, from early thymic progenitors to thymocytes that are ready to egress to the periphery (Supporting Data Values) [24]. A T-cell development scheme as well as the gating strategy are depicted in Fig. S1a and b. We performed a nonlinear dimensionality-reduction technique, UMAP, to visualize marker expression on single-cell level (Fig. S1c) and annotated the thymocyte subpopulations according to those identified by manual gating (Fig. 1a). With more than half of all living thymocytes, CD45^hi^ double-positive CD4^+^CD8^+^ (DP-II) thymocytes represent the largest subpopulation, followed by CD4^+^ single-positive (SP4) thymocytes and CD8^+^ single-positive (SP8) thymocytes [24]. UMAP visualization of exemplary thymi from all CHD subgroups shows a remarkable shift from the predominant DP cell population to more SP thymocytes which occurs with complexity of the heart disease (Fig. 1b), indicating that the frequencies of the subpopulations differ notably between acyanotic and cyanotic CHD. In acyanotic CHD, the frequency of the DP population is over 80%, whereas in the complex CHD cases it drops to about 60% (Table 2). Consequently, the next most abundant populations, which are SP4, SP8, and T-regulatory (Treg) thymocytes, are overrepresented in the latter. Also, the frequency of DP cells in the more immature CD45^lo^ thymocytes (DP-I) is lower in thymi from children with cyanotic CHD compared to thymi from children with acyanotic CHD. Concomitantly, we see higher frequencies of early thymic progenitors (ETP) and T-lineage-committed cells (TC) in the CD45^lo^ compartment in children with cyanotic CHD (Fig. 1c). Analyzing the DP cell populations in all CHD disease groups, we find a significantly reduced frequency of DP-II cells in TGA compared to all acyanotic CHD primary disease groups and in HLHS/IAA compared to VSD and cAVSD. In contrast, DP-I cells do not show significant differences among the CHD subgroups (Fig. 1d), suggesting that the loss of DP thymocytes is pronounced during later developmental stages. We used UMAP for dimensionality reduction of the cell population frequencies of all samples, including those with trisomy 21 and severe infections. The resulting UMAP representation was colored by CHD, showing thymocyte signatures in relation to primary disease group and cyanosis. Unsupervised clustering analysis of all individual patients according to the gated thymocyte subpopulations (for corresponding heatmap representation see Fig. S1d) reveals that patients with acyanotic CHD cluster in the upper part of the UMAP plot while samples from children with cyanotic CHD predominantly group in the lower part of the UMAP. Exceptions are ToF samples that can be found within both big clusters, as well as one ASD and one cAVSD sample located among the cyanotic ones (Fig. 1e). Samples from male and female children are equally distributed. In general, children with cyanotic CHD undergo surgery at earlier ages than children with acyanotic CHD (Fig. S1e), but all donors are under 1 year of age. Hierarchical clustering of all samples reveals three clusters (Fig. 1e). Cluster 1 comprises predominantly thymic samples from neonates with more complex, cyanotic CHD (ToF, TGA, HLHS/IAA). Cluster 2 comprises predominantly samples from infants with less complex and, in general, acyanotic CHD (ASD, VSD, cAVSD). Interestingly, cluster 3 comprises two samples which cluster together inside of cluster 1 and show a different composition of thymocyte subsets with remarkably low frequencies of DP thymocytes (2.3 and 0.8% DP-II thymocytes, respectively) (Figs. 1e and S1d). These thymic samples were taken from infants with TGA who additionally suffered from sepsis and necrotizing enterocolitis, respectively, and underwent surgical repair comparatively late. Most samples (8/10) from children with trisomy 21 cluster with those from patients with less complex, acyanotic CHD (Fig. 1e, Table S1). A detailed sub-analysis of the samples from children with trisomy 21 reveals higher DP cell frequencies compared to samples from children with cyanotic CHD and, concomitantly, lower SP4 and Treg frequencies. Average DP frequencies are slightly lower in trisomy 21 compared to acyanotic CHD (Fig. S2), in line with previously published results [37]. In conclusion, these data show that complex CHD is associated with a thymic signature characterized by the loss of DP thymocytes, reminiscent of thymic atrophy, and suggest that the heart defect itself influences early T-cell development.

**Table 2:** Thymocyte subsets in different CHD primary disease groups

	ASD		VSD		cAVSD		ToF		TGA		HLHS/IAA	
	Mean % (SD)											
CD45^lo^ of CD45^+^	3.80	(1.57)	4.87	(2.15)	3.72	(1.97)	5.21	(2.73)	5.58	(5.04)	3.71	(3.16)
CD45^hi^ of CD45^+^	95.95	(1.74)	94.86	(2.22)	96.04	(1.99)	94.57	(2.78)	94.16	(5.03)	96.07	(3.27)
ETP of CD45^lo^	7.29	(1.44)	10.45	(4.47)	9.08	(2.73)	10.67	(1.78)	10.68	(4.51)	22.97	(12.14)
TC of CD45^lo^	21.68	(4.56)	16.90	(6.86)	22.91	(8.34)	26.59	(14.41)	32.74	(15.32)	29.65	(13.59)
ISP4-I of CD45^lo^	50.67	(11.96)	53.71	(10.25)	49.12	(13.94)	44.27	(14.12)	43.55	(18.35)	31.64	(21.55)
DP-I of CD45^lo^	18.80	(10.02)	18.37	(8.46)	17.58	(7.91)	17.35	(11.66)	11.32	(6.61)	12.99	(6.98)
ISP4-II of CD45^hi^	1.38	(0.72)	1.34	(0.47)	1.26	(1.21)	1.24	(0.75)	1.35	(0.94)	1.42	(1.13)
DP-II of CD45^hi^	83.66	(3.53)	84.50	(2.50)	84.73	(2.98)	78.89	(6.69)	65.18	(12.62)	60.91	(21.71)
SP4 of CD45^hi^	7.91	(2.08)	8.59	(1.86)	8.31	(1.50)	11.09	(3.97)	21.28	(9.14)	22.80	(14.70)
CD4-I of SP4	64.96	(8.75)	61.45	(12.00)	55.68	(7.54)	58.04	(8.85)	49.91	(9.93)	52.26	(15.01)
CD4-II of SP4	31.34	(9.41)	31.33	(9.68)	32.62	(10.80)	36.45	(10.43)	45.30	(8.56)	39.76	(13.31)
CD4-III of SP4	3.54	(2.47)	7.02	(3.21)	11.34	(7.06)	5.35	(3.07)	4.66	(3.01)	7.73	(6.19)
SP8 of CD45^hi^	4.87	(1.94)	3.28	(1.10)	3.20	(1.41)	5.60	(2.83)	7.71	(3.34)	9.29	(5.31)
CD8-I of SP8	52.52	(13.66)	42.65	(18.47)	35.87	(5.43)	46.00	(5.58)	34.86	(11.48)	35.48	(14.83)
CD8-II of SP8	43.95	(13.62)	48.91	(14.85)	52.01	(8.58)	48.54	(7.10)	60.30	(10.56)	59.84	(14.92)
CD8-III of SP8	3.43	(1.81)	8.34	(6.49)	11.95	(6.46)	5.37	(2.22)	4.80	(2.57)	4.62	(2.42)
Treg of CD45^hi^	0.90	(0.35)	1.00	(0.24)	1.19	(0.47)	1.64	(0.68)	2.77	(1.28)	3.26	(2.24)
Treg-I of Treg	34.86	(8.29)	32.74	(12.24)	25.12	(9.76)	26.02	(6.42)	23.43	(8.25)	20.97	(10.46)
Treg-II of Treg	56.98	(10.20)	55.87	(9.91)	61.34	(12.60)	64.81	(11.86)	71.16	(7.77)	70.41	(9.77)
Treg-III of Treg	7.83	(4.97)	11.17	(3.95)	13.18	(8.34)	8.97	(6.78)	5.27	(3.75)	8.43	(6.57)
Tγδ of CD45^hi^	0.24	(0.10)	0.53	(0.45)	0.47	(0.29)	0.41	(0.45)	0.38	(0.19)	0.49	(0.25)

*CHD*, congenital heart disease; *ASD*, atrial septal defect; *VSD*, ventricular septal defect; *cAVSD*, complete atrioventricular septal defect; *ToF,* tetralogy of Fallot; *TGA*, transposition of the great arteries; *HLHS*, hypoplastic left heart syndrome; *IAA*, interrupted aortic arch; *CD*, cluster of differentiation; *ETP*, early thymic progenitors; *TC*, T-lineage-committed cells; *ISP4*, immature single-positive CD4 cells; *DP*, double-positive cells; *SP4*, CD4-single-positive cells; *SP8*, CD8-single-positive cells; *Treg*, T regulatory cells; *Tγδ*, T gamma delta cells

### High Levels of Cardiac Biomarkers Correlate with Low Frequencies of Double-Positive Thymocytes

**Fig. 2:**
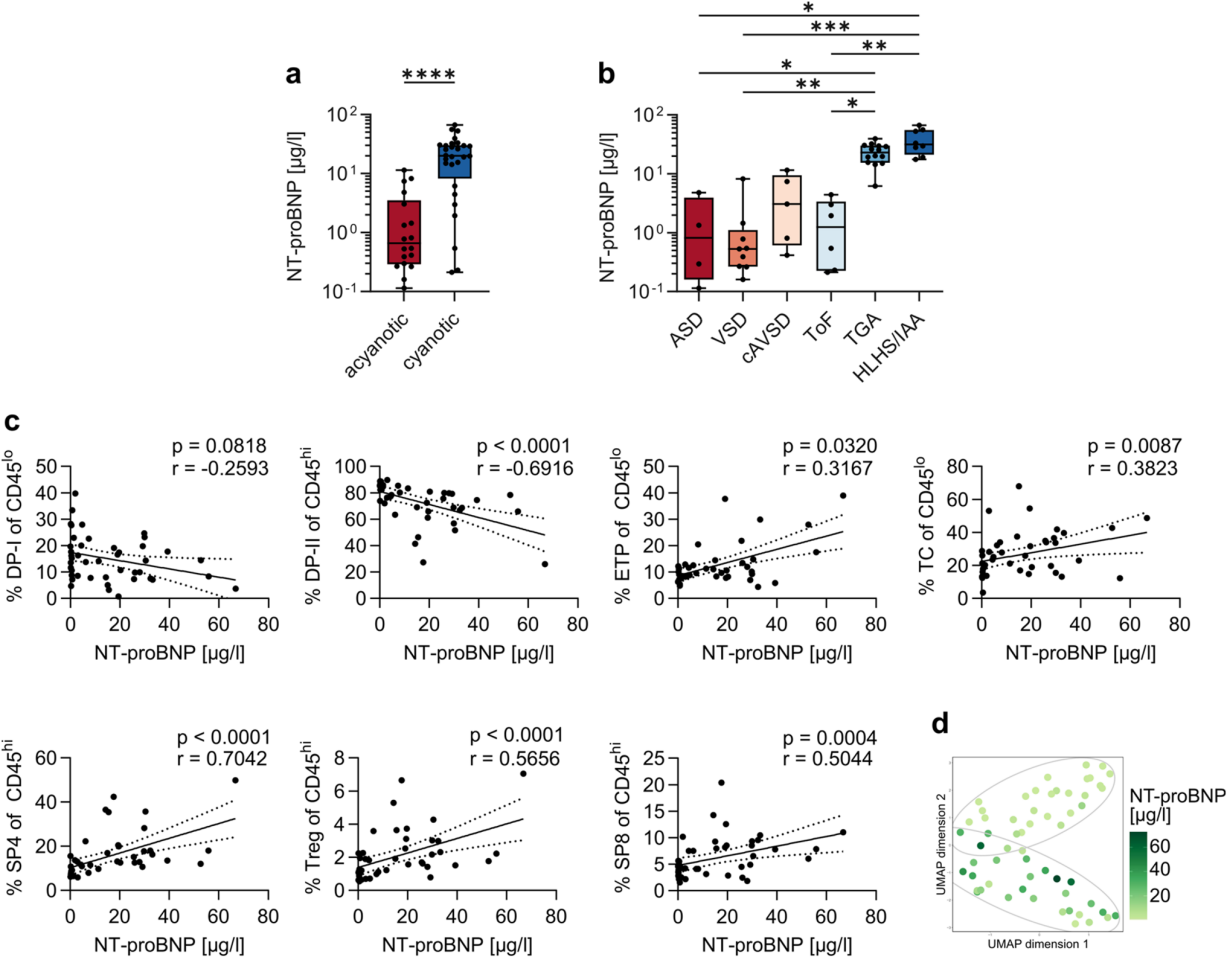
High levels of NT-proBNP correlate with depletion of double-positive thymocytes. (**a**) Levels of NT-proBNP in acyanotic and cyanotic CHD. Statistical analysis was performed with Mann–Whitney test. (**b**) Levels of NT-proBNP in the different CHD primary disease groups. Statistical analysis was performed with Kruskal–Wallis test and Dunn’s multiple comparisons test and is indicated in case of significance. (**c**) Thymocyte subsets in the context of NT-proBNP. Depicted are correlations between NT-proBNP and DP-I, DP-II, ETP, TC, SP4, Treg, and SP8 in children with CHD. Data were obtained from the cohort presented in Tables 1 and 2 (*n* = 46). Correlations were calculated with Spearman *r*. (**d**) UMAP plot showing thymocyte signature in relation to NT-proBNP indicated by color as well as clusters obtained by hierarchical clustering. UMAP representation was calculated on the data summarized in “Supporting data values” (*n* = 58 donors and 21 thymocyte subsets). Each dot represents one donor

N-terminal pro-B-type natriuretic peptide (NT-proBNP) can be used as a biomarker in heart failure [38], while Troponin T is a sensitive marker of myocardial cell injury [39] and can be used as a prognostic marker in CHD [40]. We detect significantly increased NT-proBNP levels in patients with cyanotic CHD, especially in TGA and HLHS/IAA compared to the other CHD subgroups (Fig. 2a, b). In our study population, high levels of NT-proBNP correlate strongly with low frequencies of DP-II thymocytes (*r* =  − 0.69). Concomitantly, high levels of NT-proBNP correlate with high frequencies of ETP, TC, SP4, Treg, and SP8 thymocytes. Specifically, correlation between SP4 thymocytes and NT-proBNP levels is very strong (*r* = 0.7) (Fig. 2c). We also observed significantly higher Troponin T levels in cyanotic CHD (Fig. S3a, b). High levels of Troponin T correlate strongly with low frequencies of DP-II thymocytes (*r* =  − 0.646) and high frequencies of SP4, Treg, and SP8 thymocytes (*r* = 0.661, 0.598, and 0.517, respectively) (Fig. S3c). In accordance with these findings, UMAP representation reveals a gradient from low NT-proBNP levels in cluster 2 (“acyanotic cluster”) of the UMAP (including the ToF samples located there) to high NT-proBNP levels in cluster 1 (“cyanotic cluster”) (Fig. 2d).

### Plasma Cortisol Levels Are Higher in Children with CHD Compared to Healthy Controls

**Fig. 3:**
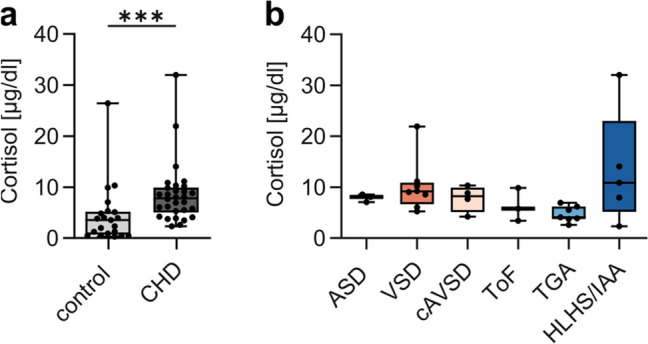
Children with CHD have higher plasma cortisol levels. Plasma cortisol concentrations of children with CHD (*n* = 30) and healthy controls (*n* = 21). One sample that was above measurement threshold was included with a cortisol level of 32 μg/dl, and three samples that were below measurement threshold were included with cortisol levels of 0.5 μg/dl. (**a**) Comparison of plasma cortisol levels of healthy controls and all CHD primary disease groups. Statistical analysis was performed using Mann–Whitney test. (**b**) Comparison of cortisol levels between CHD subgroups. Kruskal–Wallis test and Dunn’s test for multiple comparisons were performed

Glucocorticoid levels rise upon stress stimuli like psychological stress, injury, or infection, and increased glucocorticoid levels lead to a reduction in thymic size due to the loss of DP thymocytes [41, 42]. To evaluate whether high glucocorticoid levels might account for the reduced frequency of DP thymocytes in the thymi of children with complex CHD, we measured plasma cortisol levels. Children with CHD have significantly higher cortisol levels in comparison to age-matched healthy controls (median 7.8 µg/dl vs. median 3.5 µg/dl, respectively) (Fig. 3a). However, analysis of CHD subgroups shows no significant differences in cortisol levels (Fig. 3b) and cortisol levels do not correlate with the frequencies of thymocyte subpopulations (Fig. S4).

### IL-6 Is Elevated in CHD Patients with High Levels of NT-proBNP

**Fig. 4:**
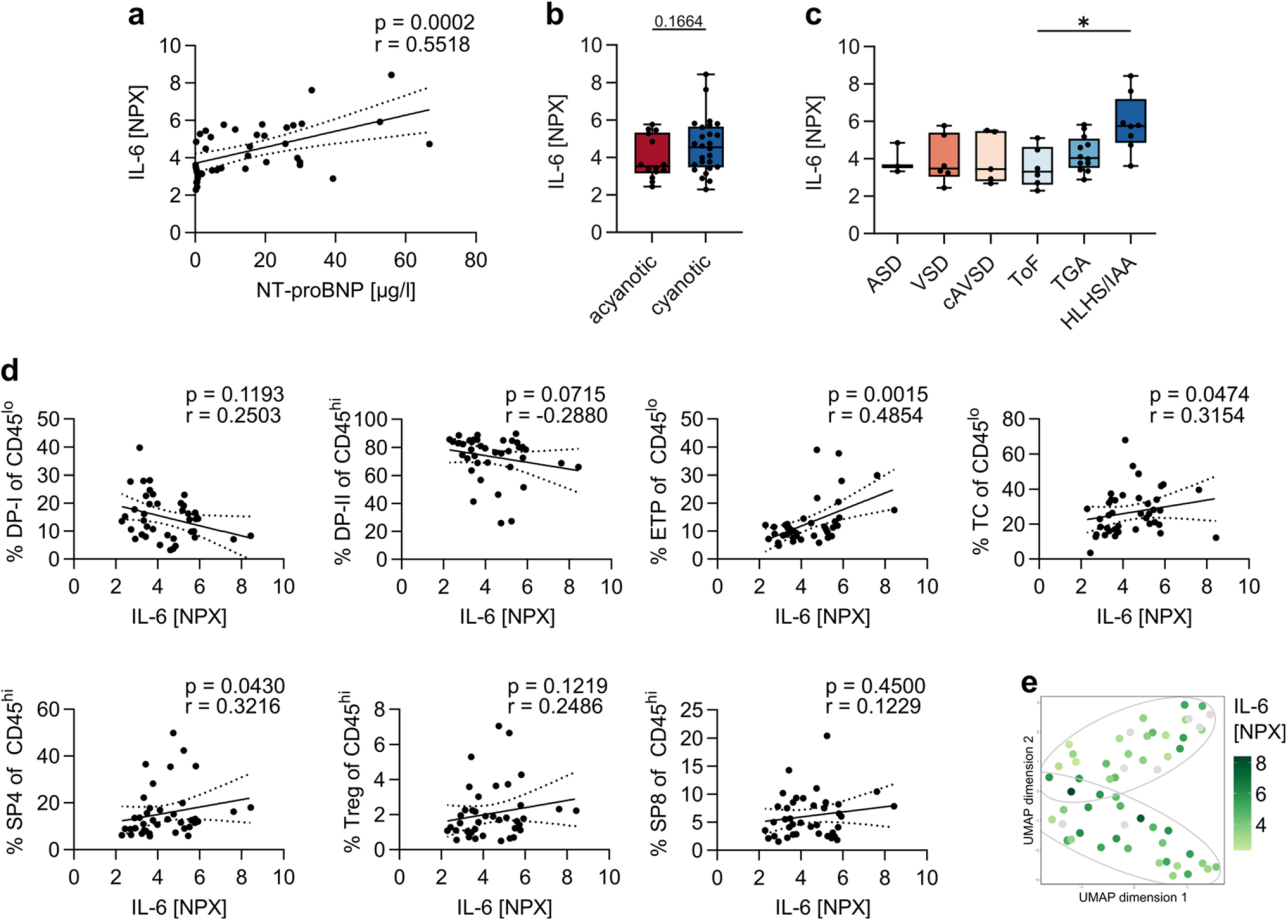
IL-6 is elevated in HLHS/IAA and correlates with high NT-proBNP levels.(**a**) Correlation between IL-6 and NT-proBNP levels in CHD patients (*n* = 40). (**b**) Levels of IL-6 in acyanotic and cyanotic CHD. Statistical analysis was performed using Mann–Whitney test. (**c**) Levels of IL-6 in the different CHD primary disease groups. Statistical analysis was performed with one-way ANOVA and Bonferroni’s multiple comparisons test and is indicated in case of significance. (**d**) Thymocyte subsets in the context of IL-6. Depicted are DP-I, DP-II, ETP, TC, SP4, Treg, and SP8 in children with CHD. Correlations were calculated with Spearman *r*. Data were obtained from the cohort presented in Tables 1 and 2 (*n* = 40). (**e**) UMAP plot showing thymocyte signature in relation to IL-6 indicated by color as well as clusters obtained by hierarchical clustering. Samples where IL-6 data are not available are depicted in gray. UMAP representation was calculated on the data summarized in “Supporting data values” (*n* = 58 donors and 21 thymocyte subsets). Each dot represents one donor

IL-6 is a pleiotropic cytokine with pro- and anti-inflammatory properties. There is increasing evidence that IL-6 plays a role in the development of cardiovascular diseases [43, 44] and can be used as a biomarker in heart failure [45]. In our study cohort, we find a strong positive correlation between levels of the cardiac biomarker NT-proBNP and IL-6 (*r* = 0.55, Fig. 4a). Our findings demonstrate significantly higher IL-6 plasma levels in children with HLHS/IAA compared to ToF. The median IL-6 level in plasma from children with cyanotic CHD is higher than in acyanotic CHD, although not significantly (Fig. 4b, c). High IL-6 levels significantly correlate with high frequencies of ETP, TC, and SP4 (Fig. 4d). In line with these results, UMAP visualization shows lower IL-6 levels in samples from children with acyanotic CHD (cluster 2) compared to samples from children with cyanotic CHD (cluster 1) (Fig. 4e). As expected, IL-6 levels were above average in the two samples from patients with severe infections. To avoid bias, those were excluded from analysis. Our data show that IL-6 is elevated in patients with the most severe CHD type and correlate strongly with NT-proBNP.

### Thymic Output Is Reduced in Cyanotic CHD

**Fig. 5:**
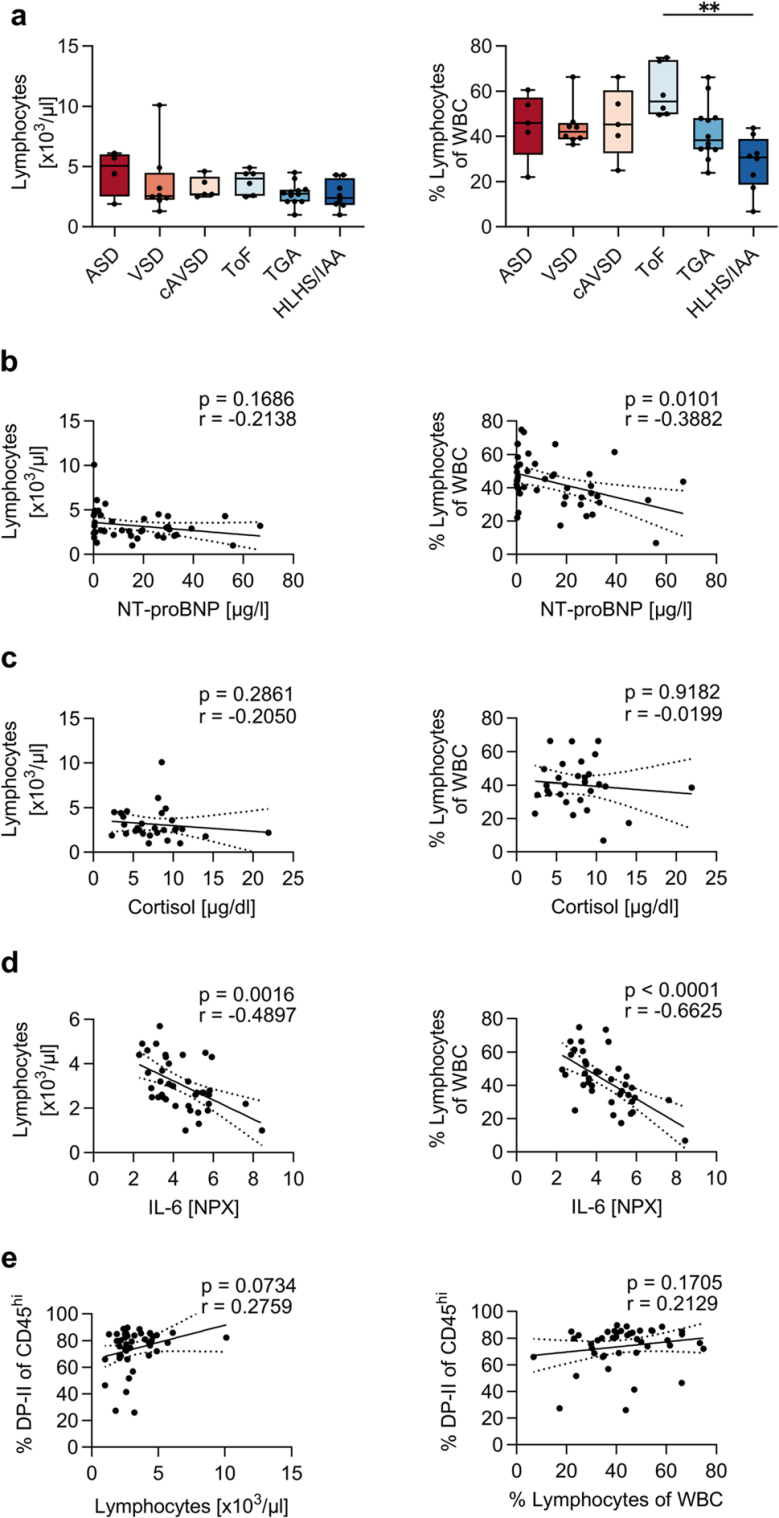
Low frequencies of lymphocytes correlate with high levels of NT-proBNP and IL-6 in peripheral blood. Numbers and frequencies of lymphocytes in the context of (**a**) CHD primary disease group (*n* = 43), (**b**) NT-proBNP (*n* = 43), (**c**) cortisol (*n* = 30), (**d**) IL-6 (*n* = 39), and (**e**) DP-II. Data were obtained from the cohort presented in Tables 1 and 2. Statistical analysis of lymphocyte numbers and frequencies in the CHD subgroups was performed with one-way ANOVA and Bonferroni’s multiple comparisons test and is indicated in case of significance. Correlations were calculated with Spearman *r*

We wondered if the thymic atrophy observed in patients with complex CHD reflects in reduced thymic output. First, we compared lymphocyte counts and frequencies in peripheral blood of our patients before thymectomy is performed. Lymphocyte counts are quite similar among CHD subgroups; however, frequencies of lymphocytes in HLHS/IAA (median 30.7%) are lower compared to ToF (median 55.5%) (Fig. 5a). There is a strong negative correlation between lymphocyte counts and IL-6 levels as well as lymphocyte frequencies and IL-6 and NT-proBNP levels, respectively. Cortisol levels and Troponin T do not correlate with lymphocyte frequencies or counts (Figs. 5b–d and S5). In addition, we see decreasing counts of lymphocytes when levels of thymic DP cells drop, indicating an influence of thymic DP depletion on the peripheral T-cell compartment (Fig. 5e).

**Fig. 6:**
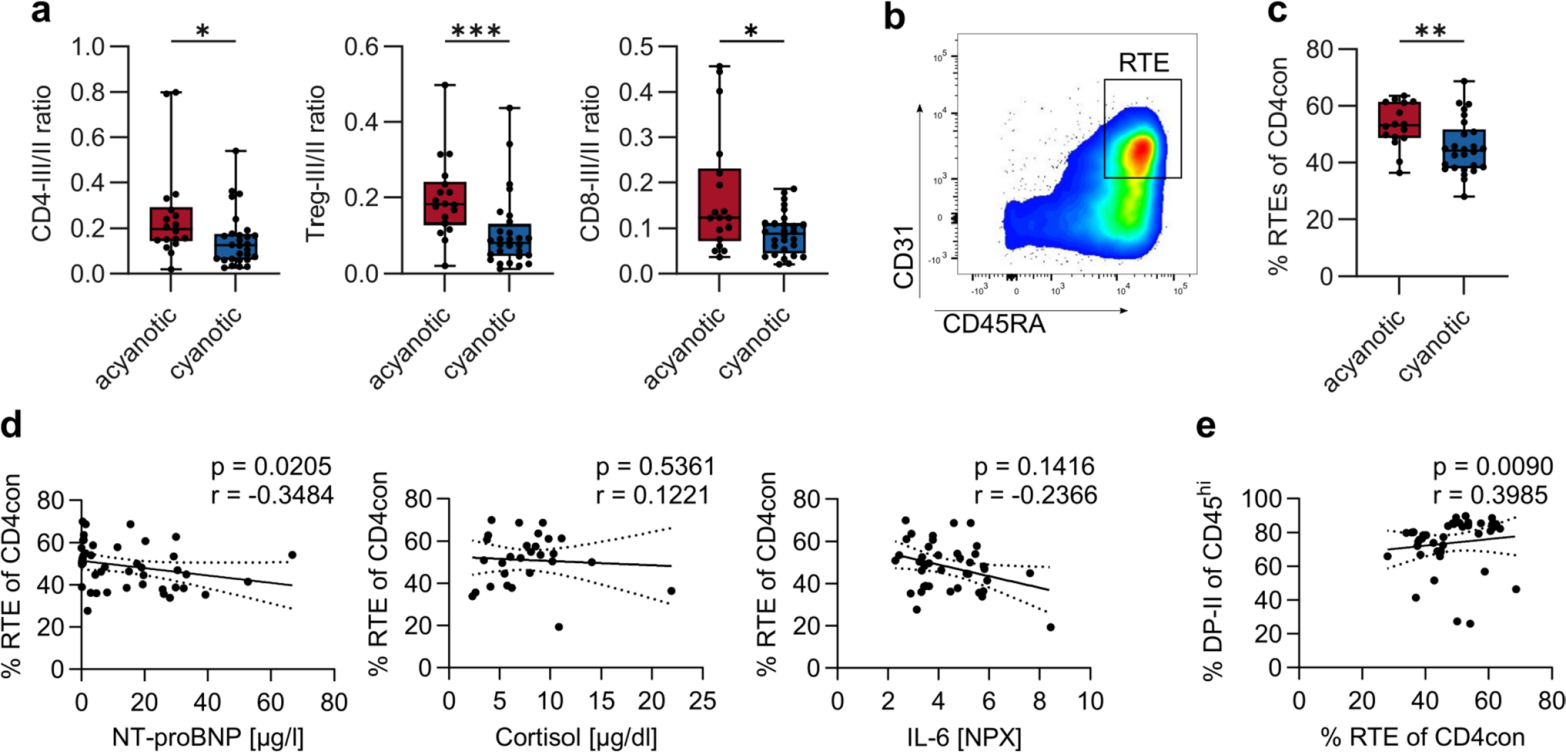
Children with cyanotic CHD show a reduced thymic output. (**a**) Ratio of the frequencies of thymocytes which are ready to egress the thymus (CD4-III, CD8-III, and Treg-III) to the other CD1a − SP populations (CD4-II, CD8-II, Treg-II) in acyanotic and cyanotic CHD (*n* = 46). Statistical analysis was performed using Mann–Whitney test. (**b**) RTE are gated as lymphocytes, CD3^+^, CD4^+^, CD4 conventional (CD25^−^CD127^−^, CD25^−^CD127^+^, CD25^+^CD127^+^), and CD31^+^CD45RA.^+^. (**c**) Frequencies of RTE in acyanotic and cyanotic CHD (*n* = 42). Statistical analysis was performed using *t*-test. (**d**) Correlation of RTE frequencies with NT-proBNP, cortisol, and IL-6. (**e**) Correlation of RTE frequencies with DP-II frequencies. Correlations were calculated with Spearman *r*. Data were obtained from the cohort presented in Tables 1 and 2

To investigate the potential impact of a compromised T-cell development on thymic output in highly complex CHD, we first analyzed the thymocytes that are ready to egress the thymus. To leave the thymus and enter circulation, thymocytes downregulate CD1a and start expressing CD45RA, the marker that characterizes naïve T cells in periphery. We distinguish the three stages of SP thymocytes according to the expression of CD1a and CD45RA: SP-I, SP-II, and SP-III thymocytes (Fig. S1a, b). The ratio of the SP-III to the SP-II thymocyte populations allows us to assess the relative abundance of thymocytes ready to egress to the periphery. In cyanotic CHD, we observed a significantly lower SP-III/SP-II ratio for CD4, Treg, and CD8 thymocytes, indicating that less mature thymocyte subpopulations are ready to leave the thymus (Fig. 6a). Next, we examined RTE present in the peripheral blood, as a measure of thymic output. CD4^+^ RTE are identified as CD4^+^ (non-Treg) CD45RA^+^CD31^+^ T cells (Fig. 6b). In children with cyanotic CHD, RTE frequencies are significantly lower compared to those with acyanotic CHD (Fig. 6c). Among CHD subgroups, frequencies of RTE do not differ (Fig. S6a). Low RTE frequencies correlate with high NT-proBNP plasma levels. There are no significant correlations between RTE frequencies and Troponin T or cortisol levels (Figs. 6d and S6b). Analysis of RTE absolute counts in peripheral blood revealed no significant differences between acyanotic and cyanotic CHD or CHD subgroups (Fig. S6c, d). Notably, we find that low frequencies of DP thymocytes correlate with low frequencies, but not absolute numbers, of RTE (Figs. 6e and S6e). In conclusion, our study demonstrates a reduced thymic output in complex CHD which reflects in lower frequencies of RTE in the periphery. In addition, high NT-proBNP levels correlate with low RTE frequencies.

## Discussion

In this study, we analyzed the impact of the cardiac defect on T-cell development in patients with CHD, a population known to be at increased risk for infections, cancer, and autoimmune diseases [16, 46–49]. Previous research has mainly linked the compromised T-cell compartment observed in these patients with early-life thymectomy [50–52]. This procedure is often necessary during corrective or palliative cardiac surgery, includes partial or total removal of the thymus, and leads to premature immune aging, characterized by T-cell lymphopenia and a progressive reduction of naïve T cells [3, 4]. Our findings challenge the conventional understanding as we observed thymic atrophy—a condition where the thymus shrinks and loses function—in patients with complex CHD even before thymectomy, suggesting that certain cardiac defects have a pivotal impact on T-cell development and thymic output. Thymus atrophy can lead to a reduced number of T cells and can result in an increased risk of infections, cancer, and autoimmune diseases [53]. This highlights the need for a comprehensive investigation of the immune status in patients with CHD, considering both the impact of the heart disease and, if applicable, thymectomy. Moreover, the population of adults with CHD (ACHD) in Europe is increasing steadily, numbering 2.3 million at present. The majority of ACHD suffers from non-cardiac comorbidities such as infectious diseases, neoplasms, or endocrine and metabolic conditions, and the extent of CHD complexity is closely correlated with the prevalence of these comorbidities [54, 55]. Therefore, it is essential to understand the potential influence of immunological abnormalities on the health of CHD patients, independently of those resulting from thymectomy.

To investigate the interrelation of the cardiac defect and immune function, we analyzed the early T-cell compartment in 58 infants with CHD. We found thymic atrophy, characterized by a depletion of DP thymocytes, predominantly in patients with complex and cyanotic CHD, including TGA and HLHS or IAA. Conversely, patients with mild or acyanotic forms of CHD showed normal DP cell frequencies. A noteworthy case is ToF, the most common form of cyanotic CHD in children who have survived untreated beyond neonatal age [32]. While most ToF patients have sufficient pulmonary blood flow at birth, surgical intervention becomes necessary within the first year of life when cyanosis develops [32]. Despite its classification as cyanotic CHD, our investigations revealed that most ToF patients showed normal thymic function without the signs of thymic atrophy seen in children with cyanotic CHD.

Based on cluster analysis of thymocyte populations, we identified three distinct groups of patients, with two large clusters primarily comprising either acyanotic or cyanotic patients. ToF cases were present in both clusters, emphasizing the unique nature of this heart condition within our study cohort. Our findings on thymic atrophy are supported by prior reports describing histomorphological alterations in the thymi of CHD patients. Specifically, patients with cyanotic CHD exhibited changes in Hassall’s corpuscles, cortical starry sky appearance, effacement of the corticomedullary junction, and cortical thinning, all features of thymus involution, and attributed to thymocyte loss [56–58]. Our study provides new insights on thymic atrophy in patients with complex CHD at the cellular level, supporting the described histomorphological features by demonstrating the pronounced loss of DP cells.

Thymic atrophy may be attributed to low oxygen levels, which lead to the expression of hypoxia-inducible factor 1-alpha (HIF-1α). This crucial transcription factor adapts cellular responses to oxygen availability; induces glycolysis, angiogenesis, and cell growth or death; and also regulates T-cell differentiation and function [57, 59]. A previous study showed increased expression of HIF-1α in the thymus of individuals with cyanotic CHD, representing a potential mechanism contributing to thymic atrophy in this patient subgroup [57]. For future studies, additional investigations should aim to decipher whether HIF-1α or HIF-1α-induced genes exhibit differential expression across different thymic cell populations derived from various CHD. If HIF-1α expression contributes to thymic atrophy, targeting its function could be explored as a potential treatment strategy [60].

Genetic abnormalities, found in approximately 10% of CHD cases, may also contribute to the observed thymic atrophy [61]. Some of these CHD-associated genes are not only essential for heart morphogenesis but also play a critical role in thymic development (e.g. CHD7, FOXN-1, GATA4, JAG1, NKX2, TBX1) [36, 62–67].

Embryonic development of the thymus and heart are closely connected; they both arise from adjacent tissues and share common progenitor cells, such as neural crest cells (NCC). NCC, a multipotent cell population stemming from the dorsal neural tube, are essential for normal cardiovascular development, but also comprise the capsule and pericytes of the thymus [69, 70]. Loss or dysregulation of NCC signaling pathways (e.g., Notch, Bmp, Wnt) can lead to the development of CHD and/or result in a hypoplastic thymus [69, 71]. Mutations in genes critical for thymic development can cause inborn errors of immunity (IEIs), often characterized by immunodeficiency [72]. Early embryonic defects in the fourth branchial arch, as observed in conditions like DiGeorge or DiGeorge-like syndromes, showcase the interplay between cardiac and immune development [73]. DGS, frequently associated with a 22q11.2 deletion, is a highly variable disease and often characterized by cardiac abnormalities, thymic hypoplasia, and mild to severe T-cell immunodeficiency [74, 75]. Haploinsufficiency of *TBX1*, a key player in DGS, contributes to cardiovascular and thymic defects and controls the expression of approximately 2000 genes through epigenetic modifications [72, 74–76]. This further emphasizes a possible role of epigenetic mechanisms in the intricate interplay among genetic factors, cardiac development, and immune function in children with complex CHD. Likewise, Down syndrome, resulting from trisomy of chromosome 21, is another genetic condition highly prevalent in the pediatric CHD population. Up to 50% of individuals with trisomy 21 have some type of CHD, most commonly acyanotic AVSD or VSD, and also show thymic abnormalities [37, 68]. However, observations from our study cohort do not indicate that trisomy 21 leads to thymic atrophy resulting from a significant depletion of DP thymocytes. Instead, our analysis of patients with trisomy 21 reveals a modest decrease in DP thymocytes and a trend toward elevated frequencies of SP4 and SP8 cells, suggesting that the impact of CHD on thymic function may be more pronounced than that observed in trisomy 21.

Prenatal exposure to risk factors, such as maternal medications, cigarette smoking, pregestational diabetes, malnutrition, or in utero infections, is associated with the development of CHD in the fetus [77–80] and also linked to thymic atrophy in utero [81–84]. Other risk factors, such as pre- and postnatal infections, can have a significant impact on thymic function, as demonstrated by two patients in our study. The clustering algorithm identified these two patients as an independent cluster, with the most remarkable feature being the nearly complete absence of DP thymocytes. Both children were born with TGA and experienced illness during the early postnatal period, specifically necrotizing enterocolitis (NEC) and sepsis. It is well known that the thymus is highly vulnerable to insults, such as infections, but a massive depletion of DP thymocytes has only been observed in murine models of sepsis [5, 85, 86]. Our study documents for the first time at a cellular level the dramatic thymic atrophy in human neonates with NEC or sepsis, in line with a previous study showing low thymocyte numbers in a patient with protracted rotavirus infection [87]. Infection-induced thymic atrophy can be caused by the activation of the hypothalamus–pituitary–adrenal (HPA) axis and the release of glucocorticoids [5]. Other stressors, such as heart surgery or hypoxia, may also lead to the activation of the HPA axis and increased production of cortisol, which may contribute to thymic atrophy and compromised thymic function [41, 88]. In our study, we analyzed morning cortisol plasma levels in pediatric CHD patients compared to age-matched non-CHD controls. The CHD group showed higher cortisol levels, suggesting an increased stress response in infants requiring heart surgery. However, we did not observe significant differences in cortisol levels among the different disease groups, in line with a previous report showing similar salivary cortisol levels in acyanotic and cyanotic children with CHD [89]. These findings suggest that the loss of DP thymocytes in pediatric patients with complex CHD is not exclusively driven by stress-induced mechanisms. While our findings are based on plasma cortisol levels, it is important to consider the potential influence of elevated intrathymic cortisol levels acting on DP thymocytes in cases of complex CHD. Local synthesis of glucocorticoids has been shown to impact thymocyte development and negative selection [90, 91]. Further research is needed to decipher intrathymic cortisol levels and their potential implications for thymic atrophy in individuals with CHD.

Besides glucocorticoids, also circulating inflammatory markers might contribute to a compromised adaptive immunity in CHD patients. Previous studies have described elevated levels of IL-6 in both serum and myocardium of infants with cyanotic CHD [44, 92–94]. In agreement with these data, we observed higher IL-6 levels in patients with elevated NT-proBNP levels. However, our data imply that IL-6 and NT-proBNP can only serve as markers for thymic atrophy rather than being causative factors for DP depletion. In general, IL-6 promotes differentiation of naïve T cells into proinflammatory Th17 cells and inhibits Treg function and expansion [95, 96]. Under lymphopenic conditions, IL-6 may facilitate lymphopenia-induced proliferation of autoreactive T cells [95, 97], which could contribute to the development of co-morbidities observed in adult CHD patients [54, 55].

The compromised T-cell development in patients with complex CHD is reflected in their peripheral immune cell compartment. We show that patients with pronounced thymic atrophy exhibit low numbers of lymphocytes and RTE, a subset of naïve T cells that have most recently completed intrathymic development and entered the lymphoid periphery. RTE closely correlate with TREC levels, which are accessed during newborn screening programs for severe combined immunodeficiency (SCID) [20, 98, 99]. Recently, lower TREC levels have been described in newborns with CHD, suggesting that the heart defect is associated with the reduced thymic output [100, 101]. Although a direct comparison is complicated by the use of diverse CHD classification criteria, the data suggest that patients with complex CHD have the lowest neonatal TREC levels [100, 101]. Reduced thymic output is a common cause of T-cell lymphopenia which has been associated with an increased risk to develop autoimmune diseases [20, 102]. Early-life thymectomy may exacerbate T-cell lymphopenia and further impact the health of patients with CHD [46]. A recent retrospective study has demonstrated that thymectomy in adults leads to reduced T-cell production, a cytokine signature of immune dysregulation and a significantly increased mortality and cancer risk, underscoring the importance of preserving the thymus at any age [103]. Consequently, the removal of the thymus shortly after birth in the context of congenital heart disease could pose even greater risks, emphasizing the need to develop surgical techniques to prevent routine total thymectomy and, instead, preserve at least parts of the thymus. This is particularly crucial in patients with complex CHD, as their thymic output is notably reduced from birth.

The present study provides important insights on the compromised T-cell development observed in patients with complex forms of CHD. However, there are also limitations to be considered. Our analysis was limited to thymus samples obtained from CHD patients, preventing a direct comparison with thymic tissue from healthy donors, which for obvious reasons is not readily accessible. In addition, the variability within individual disease groups suggests the influence of comorbidities, severe postnatal illness, and genetic or epigenetic effects on thymus health. To address these limitations, future investigations should focus on the identification of biomarkers that can aid in stratifying patients with complex CHD who exhibit a compromised T-cell development. Understanding the impact of CHD on thymus health is crucial for the accurate interpretation of investigations conducted on human thymus samples and to ensure that CHD patients receive appropriate care from healthcare providers.

### Supplementary Information

Below is the link to the electronic supplementary material.

Supplementary file1 (PDF 3.62 MB)

Supplementary file2 (XlSX 35.1 KB)

## Data Availability

All data in this study are presented in the article or supplementary information.

## Abbreviations

ASD: Atrial septal defect
cAVSD: Complete atrioventricular septal defect
CHD: Congenital heart disease
DP: Double-positive CD4^+^CD8^+^
ETP: Early thymic progenitors
HLHS: Hypoplastic left heart syndrome
IAA: Interrupted aortic arch
ISP: Immature single-positive
NT-proBNP: N-terminal pro-B-type natriuretic peptide
RTE: Recent thymic emigrants
SCID: Severe combined immunodeficiency
SP: Single-positive
TC: T-lineage-committed
TGA: Transposition of the great arteries
ToF: Tetralogy of Fallot
TREC: T-cell receptor excision circles
Treg: T-regulatory cells
VSD: Ventricular septal defect

## Code Availability

All code for analysis is available upon request.
