# Supplementary material for: Thymic Atrophy and Immune Dysregulation in Infants with Complex Congenital Heart Disease
Source: J Clin Immunol. 2024 Feb 23;44(3):69. doi: 10.1007/s10875-024-01662-4 (PMC10891212; doi:10.1007/s10875-024-01662-4)
Supplement: Supplementary file 1 — Supplementary file1 (PDF 3.62 MB) [file 10875_2024_1662_MOESM1_ESM.pdf]

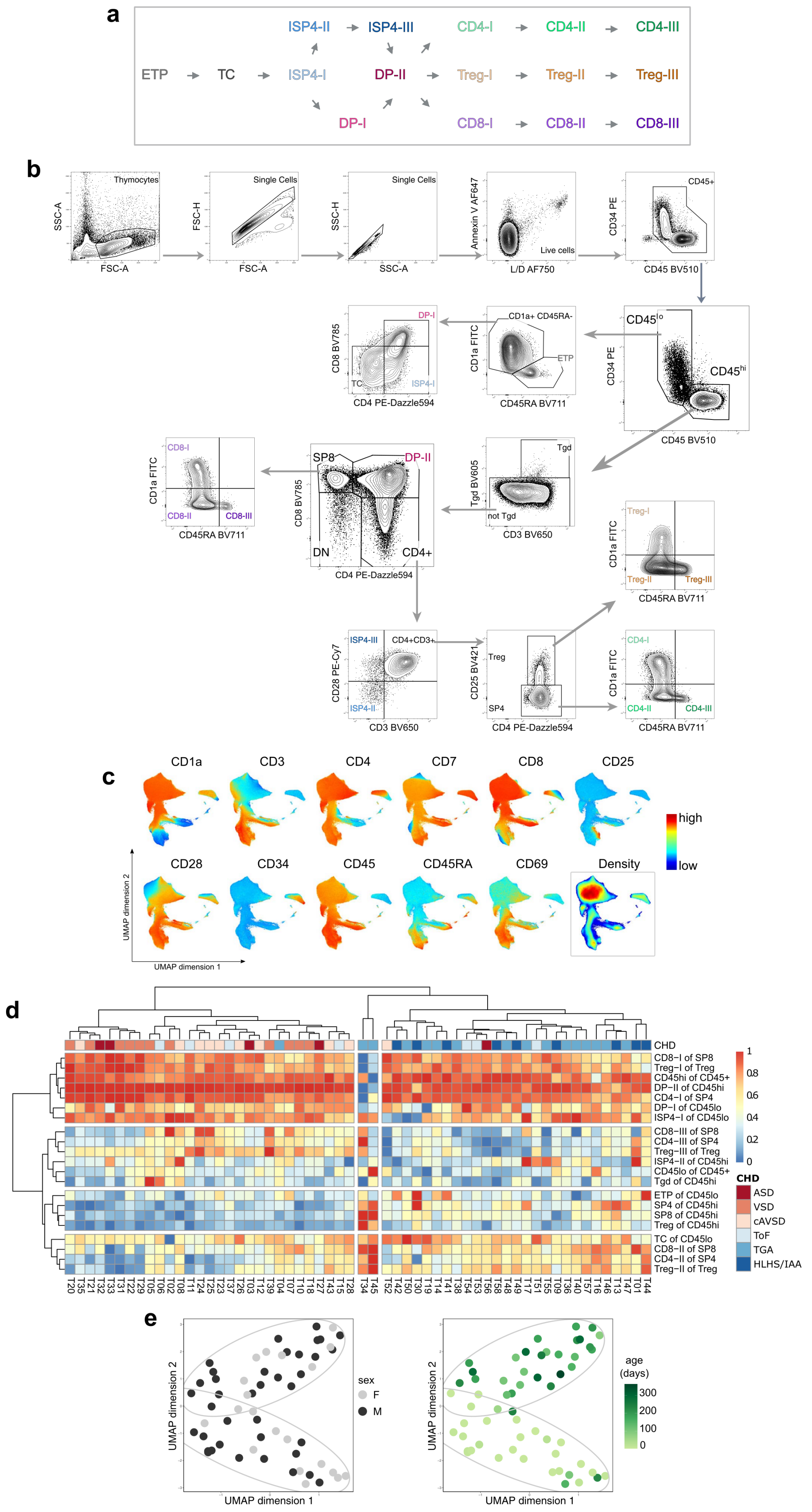

Figure S1. (a) T cell development scheme. **(b)** Gating strategy for immunophenotypic analysis of thymocytes. The figure shows a representative sample of a donor with ToF. **(c)** UMAP plots of live CD45+ cells color coded by the expression of cell surface markers and cell density. Plots show the merged cells from one exemplary donor per CHD primary disease group. Each dot represents one cell. **(d)** Heatmap of frequencies of cell populations forming the thymocyte signature. Population frequencies in columns are scaled logarithmically and are individually normalized to the range of 0-1. CHD primary disease group is annotated for each sample. **(e)** UMAP plots showing thymocyte signatures in relation to sex and age indicated by color as well as clusters obtained by hierarchical clustering. UMAP representation was calculated on the data summarized in “Supporting Data Values” (n=58 donors and 21 thymocyte subsets). Each dot represents one donor.

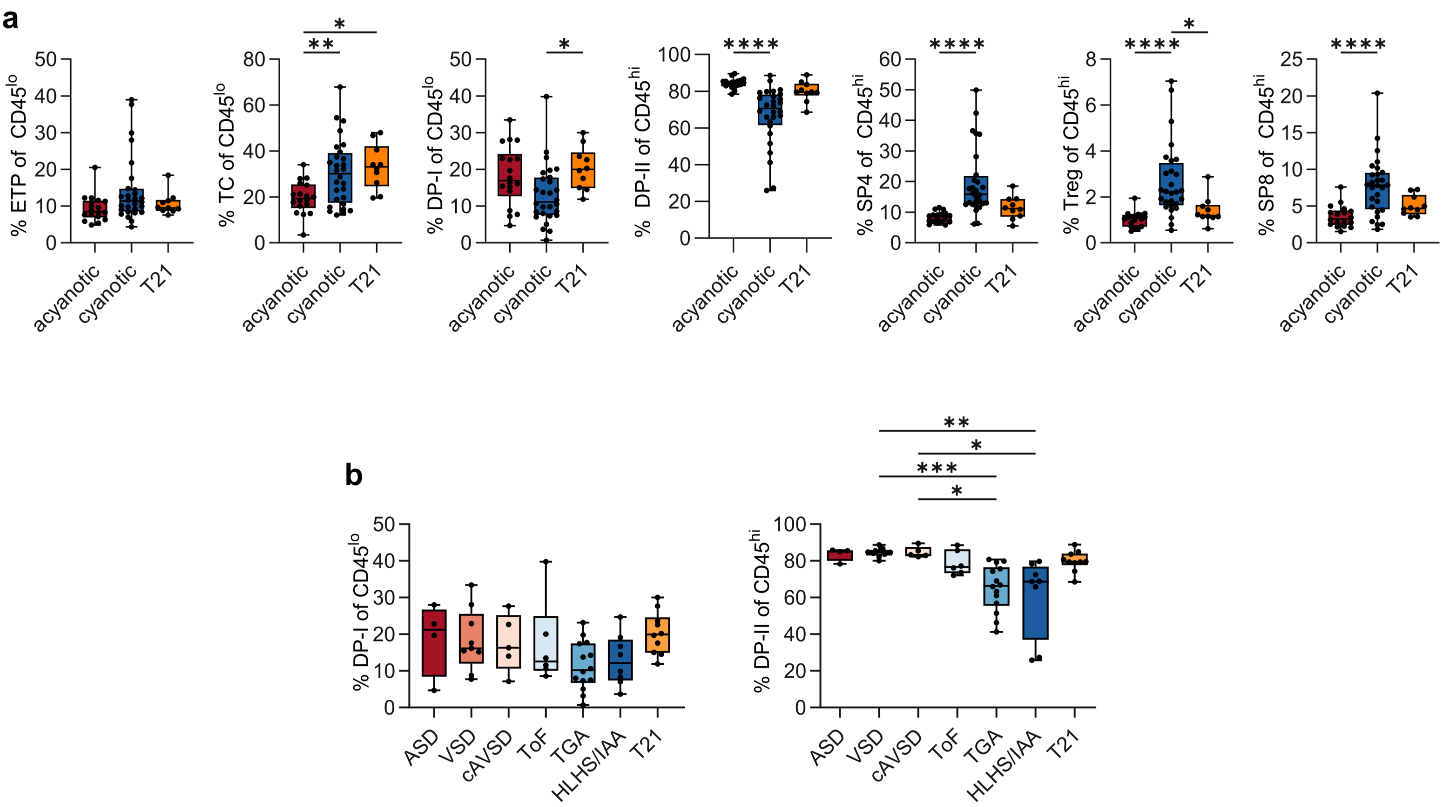

Figure S2. (a) Comparison of thymocyte subpopulations in acyanotic CHD, cyanotic CHD and trisomy 21 (T21). **(b)** Comparison of the thymocyte subsets DP-I and DP-II between the CHD groups and T21. Statistical analysis was performed with one-way ANOVA and Bonferroni's multiple comparisons test and Kruskal-Wallis test and Dunn´s multiple comparisons test, respectively, and is indicated in case of significance. Data were obtained from the cohort presented in Supplementary Table 1 (n=10).

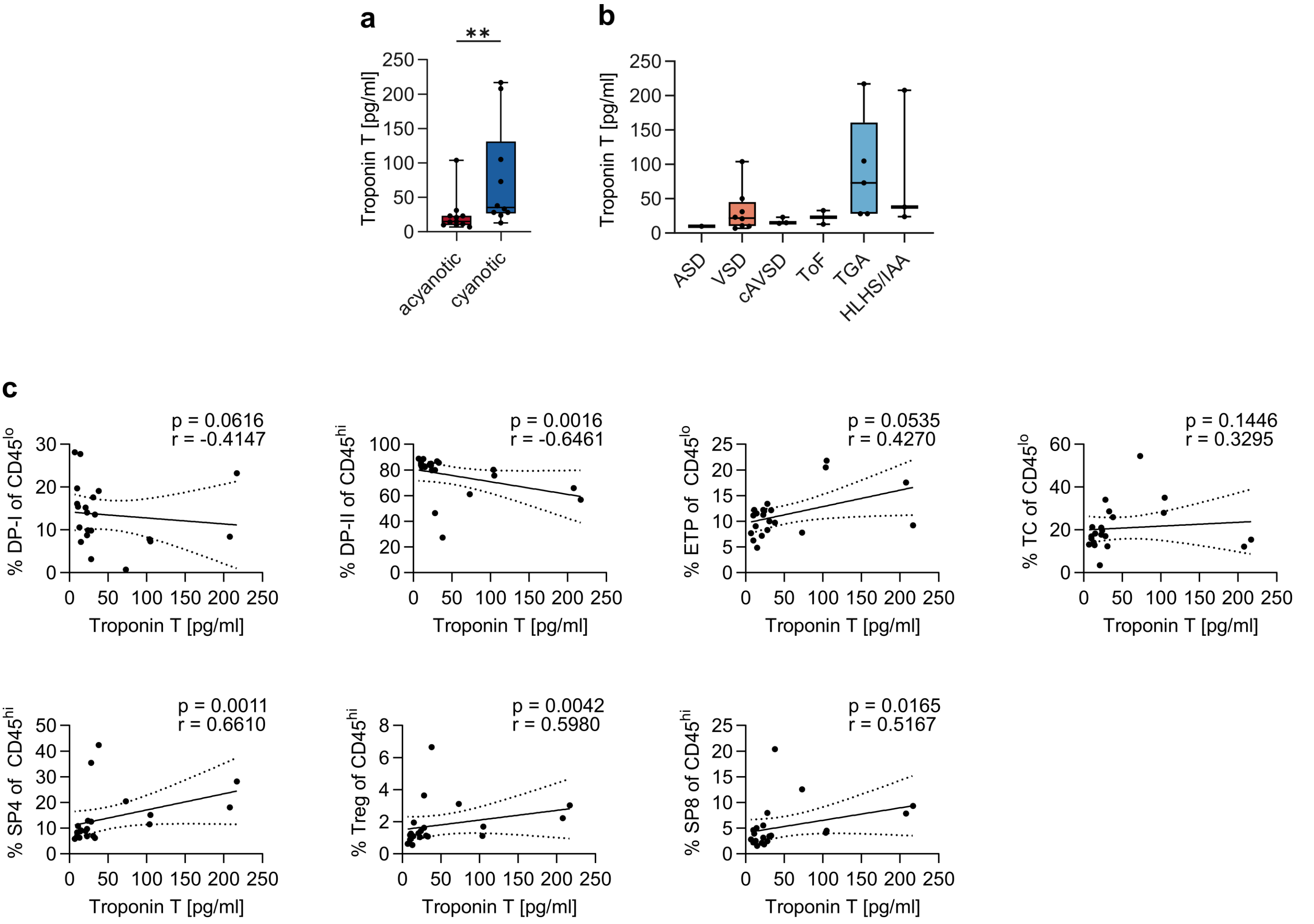

Figure S3. (a) Levels of Troponin T in acyanotic and cyanotic CHD. Statistical analysis was performed with Mann-Whitney test. **(b)** Levels of Troponin T in the different CHD primary disease groups. Statistical analysis was performed with Kruskal-Wallis test and Dunn´s multiple comparisons test and is indicated in case of significance. **(c)** Thymocyte subsets in the context of Troponin T. Depicted are ETP, TC, DP-I, DP-II, SP4, Treg and SP8 in children with CHD. Data were obtained from the cohort presented in Table 1 (n=21). Correlations were calculated with Spearman r.

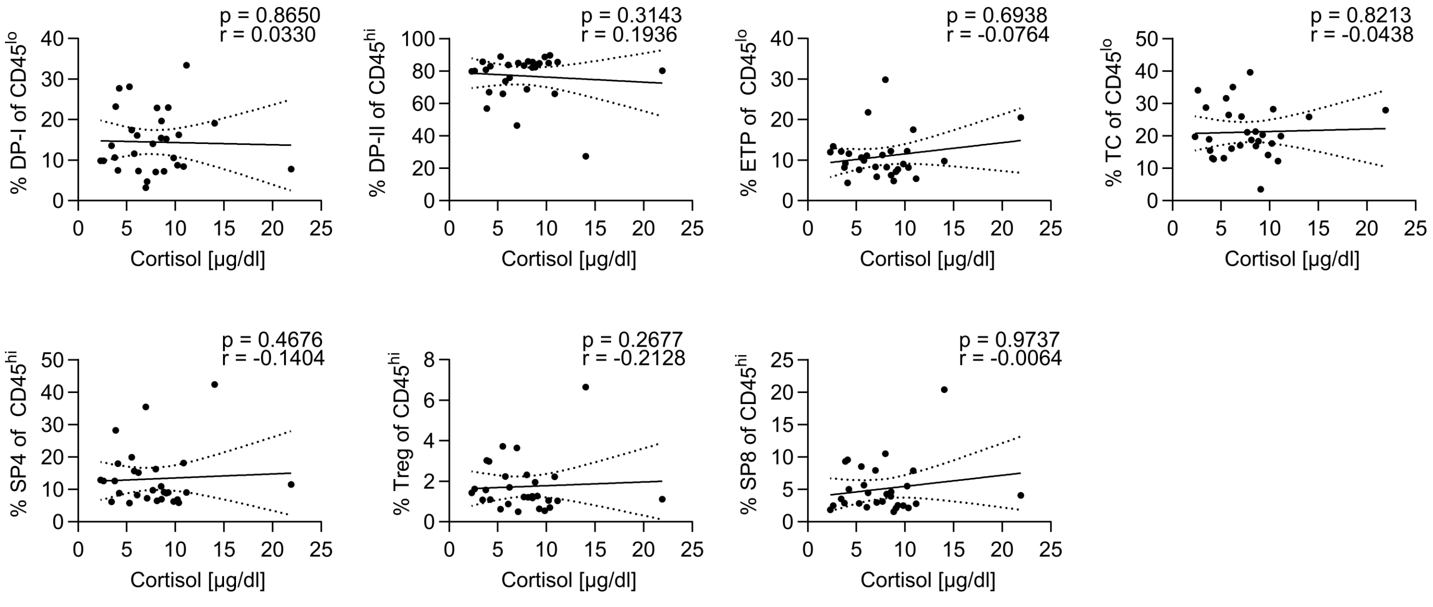

Figure S4. Thymocyte subsets in the context of cortisol levels. Depicted are correlations between cortisol and ETP, TC, DP-I, DP-II, SP4, Treg and SP8 in children with CHD aged 2 to 363 days (n=30). Correlations were calculated with Spearman r.

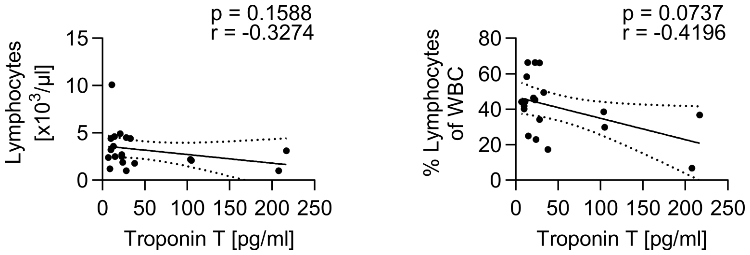

Figure S5. Correlations of lymphocytes numbers and frequencies with Troponin T. Data were obtained from the cohort presented in Table 1 (n=19). Correlations were calculated with Spearman r.

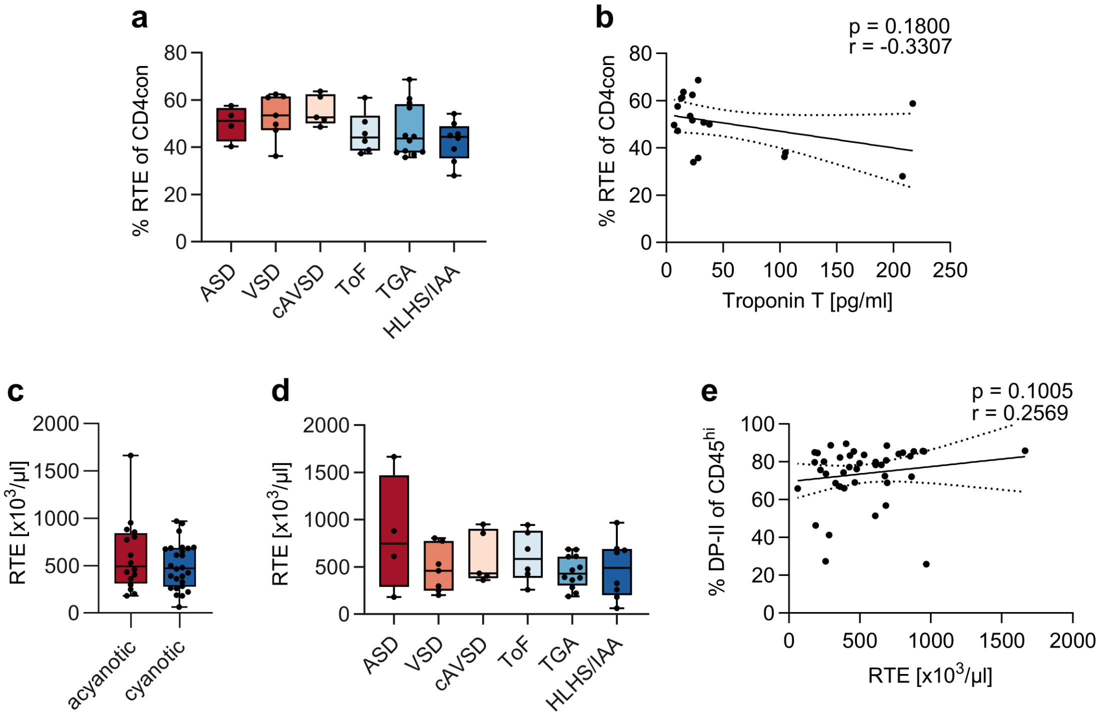

Figure S6. (a) Frequencies of RTE in the different CHD primary disease groups (n=42). Statistical analysis was performed with Kruskal-Wallis test and Dunn´s multiple comparisons test. **(b)** Correlation of RTE frequencies with Troponin T (n=18). **(c)** Absolute numbers of RTE in acyanotic and cyanotic CHD (n=42). Statistical analysis was performed using Mann-Whitney test. **(d)** Absolute numbers of RTE in the different CHD primary disease groups. Statistical analysis was performed with one-way ANOVA and Bonferroni's multiple comparisons test. **(e)** Correlation of RTE absolute numbers and DP-II frequencies. Correlations were calculated with Spearman r.

SUPPLEMENTARY TABLE 1. Patient characteristics of subgroup with trisomy 21

	n	lymphocytes in peripheral blood [10^9/l] (n=9) count (% of WBC)		NT-proBNP [ng/l] (n=10) mean (SD)		Troponin T [pg/ml] (n=7) mean (SD)	
all	10	2.24	(39.21)	3812	(6762)	41	(45)
**Age**							
< 30 days	1	n/a	n/a	22523	n/a	n/a	n/a
30-180 days	6	2.20	(37.88)	1827	(1516)	54	(49)
> 180 days	3	2.33	(41.87)	1544	(2334)	10	(1)
**Sex**							
female	5	2.20	(44.63)	5276	(9716)	55	(71)
male	5	2.28	(34.88)	2347	(1770)	31	(21)
**CHD primary diagnosis**							
ASD	1	3.20	(46.00)	261	n/a	10	n/a
VSD	2	2.75	(44.80)	2567	(2364)	50	n/a
cAVSD	5	2.06	(40.10)	2013	(1616)	55	(56)
ToF	1	1.20	(16.80)	134	n/a	9	n/a
TGA	0	n/a	n/a	n/a	n/a	n/a	n/a
HLHS/IAA	1	n/a	n/a	22523	n/a	n/a	n/a
**Cyanosis**							
no	8	2.38	(42.01)	1932	(1676)	46	(47)
yes	2	1.20	(16.80)	11329	(15831)	9	n/a
**Prematurity**							
no	8	2.11	(38.19)	4273	(7533)	46	(53)
yes	2	2.70	(42.80)	1965	(2409)	29	(26)

CHD, congenital heart disease; *ASD*, atrial septal defect; *VSD*, ventricular septal defect; *cAVSD*, complete atrioventricular septal defect; *ToF*, tetralogy of Fallot; *TGA*, transposition of the great arteries; *HLHS*, hypoplastic left heart syndrome; *IAA*, interrupted aortic arch; *NT-proBNP*, N-terminal pro-B-type natriuretic peptide; *SD*, standard deviation; *WBC*, white blood cells.
